# Why do women with melanoma do better than men?

**DOI:** 10.7554/eLife.33511

**Published:** 2018-01-16

**Authors:** Keiran SM Smalley

**Affiliations:** 1 Department of Tumor BiologyThe Moffitt Cancer Center & Research InstituteTampaUnited States; 2 Department of Cutaneous OncologyThe Moffitt Cancer Center & Research InstituteTampaUnited States

**Keywords:** GPER, GPCR, melanoma, Human, Mouse

## Abstract

Harnessing female sex hormones may improve how all patients with melanoma respond to treatment.

**Related research article** Natale CA, Li J, Zhang J, Dahal A, Dentchev T, Stanger BZ, Ridky TW. 2018. Activation of G protein-coupled estrogen receptor signaling inhibits melanoma and improves response to immune checkpoint blockade. *eLife* **7**:e31770. doi: 10.7554/eLife.31770

Melanoma is a deadly form of skin cancer that develops from pigment-producing cells called melanocytes. Men have a higher risk of developing this cancer than women, and a worse prognosis if they do. It has been suggested that social issues underlie this gender difference because, historically, men tended to work outdoors more than women, and thus received more exposure to the sun (which is the major risk factor for melanoma). Men are also less likely to visit a doctor and get suspicious-looking skin lesions examined. However, even when these and other similar factors are accounted for, men with melanoma still have poorer outcomes than their female counterparts (Joosse et al., 2013) – meaning something else must be happening as well.

It has long been suspected that female sex hormones are linked to the control of skin pigmentation. Indeed, observers as far back as Hippocrates noted that pregnancy was often associated with changes in skin coloration. Now, in eLife, Todd Ridky and colleagues from the University of Pennsylvania – including Christopher Natale as first author – report how the female sex hormone estrogen affects melanoma development and response to therapy (Natale et al., 2018).

Natale et al. started by growing a skin-like tissue that contained human melanocytes with a form of the cancer-causing oncogene BRAF that could be induced by a chemical called doxycycline (Wellbrock et al., 2004). This humanized skin was grafted onto the backs of mice that were then separated into two groups. One group was allowed to breed and the other was not. The mice were then fed doxycycline in their drinking water to activate the BRAF oncogene. After a period of 15 weeks, and three consecutive pregnancies in the breeding group, the humanized skin was collected from the mice and analyzed. In the non-breeding mice, the skin contained melanomas, but the skin from the pregnant mice did not and looked mostly normal.

Clearly the pregnancies had prevented melanoma development, but how? One clue came from the mouse skin of the breeding mice, which was highly pigmented – despite containing fewer melanocytes than the non-breeding mice. Under normal conditions, melanocytes protect the cells of the skin from the damaging effects of ultraviolet radiation (UV) by producing a UV-absorbing pigment called melanin (Haass et al., 2005). Melanocytes must be both activated and differentiated to produce the pigment, and the darkening of the skin in the breeding mice indicated that these cells were highly differentiated.

To determine if female sex hormones were behind the enhanced differentiation of the melanocytes, Natale et al. next tested how estrogen and progesterone affected pigment production. They found that estrogen induced pigmentation of the melanocytes, whereas progesterone did not. However, estrogen was not signaling through its classical receptors because these receptors are not found in melanocytes and melanoma (Figure 1). Instead, it turned out that estrogen was signaling through a different receptor called the G-protein coupled estrogen receptor (GPER; Natale et al., 2016). The fact that estrogen was not signaling through its usual receptor offered the potential to therapeutically target this more unusual pathway without any of the side effects normally associated with using estrogen treatments.

**Figure 1.**
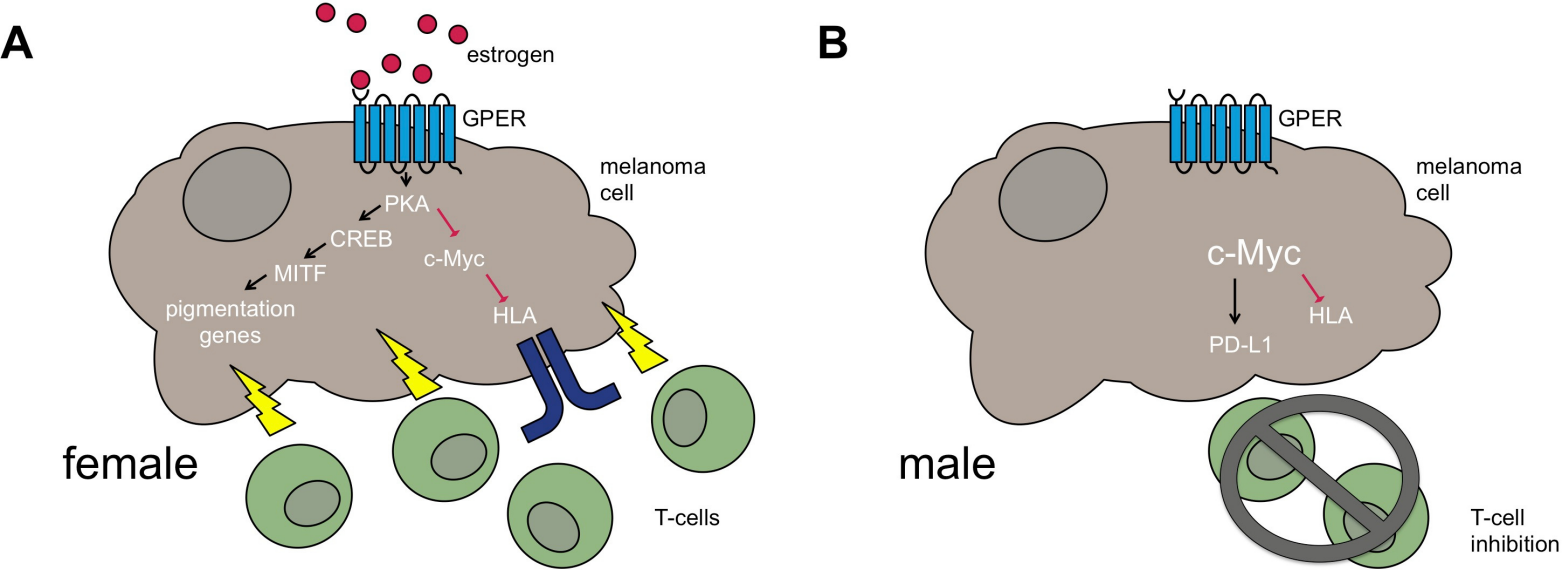
Differences between female and male melanoma patients. (**A**) In female melanoma patients, the hormone estrogen (red circles) activates the G-protein coupled estrogen receptor (GPER, light blue), which increases signaling through the kinase PKA and the transcription factor CREB. This leads to increased expression of another transcription factor MITF, which in turn switches on pigmentation genes (and other genes responsible for melanocyte differentiation). At the same time, PKA suppresses a third transcription factor c-Myc, meaning that it no longer inhibits the expression of the cell-surface proteins HLA (blue). The net result is the increased recognition of the melanoma by the immune system (T-cells, shown in green). (**B**) Men have less estrogen, and so the GPER is not activated and c-Myc levels are likely to be higher. The lack of active GPER means that the pigmentation genes are not as active as they are in females. The high levels of c-Myc also inhibit the production of HLA and activate the immune checkpoint activator PD-L1. Together these factors lead to the melanoma adopting a less differentiated state and to decreased immune function via T-cell inhibition.

Natale et al. next showed that both estrogen and a drug called G-1 (which activates GPER) inhibited the growth of melanoma cells, irrespective of which oncogene was driving them. From a functional standpoint, the researchers also found that the activation of GPER destabilized a transcription factor called c-Myc that regulates various genes that are involved in cell growth, differentiation and immune checkpoints (Dang, 2012; Figure 1). Immune checkpoint inhibitors, including nivolumab, atezolizumab and ipilimumab, are some of the most exciting new melanoma therapies. These so-called immunotherapies work by blocking the inhibitory signals that would otherwise prevent the immune system from recognizing the tumor. Relief of this inhibition unleashes the full power of the immune system, which often keeps the patient’s cancer under control for prolonged periods (Larkin et al., 2015).

Despite the success of many immunotherapies, they are not effective in around 50% of melanoma patients. To address whether GPER activation could improve the response to immunotherapy, Natale et al. performed experiments in which melanoma cells were treated with either the GPER-activating drug or a control solution before they were injected into the mice. They found that the mice with tumors in which the GPER was activated responded better to immunotherapy than those without the GPER treatment. Similar results were seen when the mice were treated concurrently with the GPER activator and an immunotherapy. The activation of GPER worked by increasing the number of active immune cells that trafficked into the tumors (Figure 1).

It seems likely that female melanoma patients often have better outcomes because estrogen increases the immune response against melanomas. But could men also benefit from these new findings? There is already evidence that some men may experience the benefits of estrogen on their melanomas. Recent studies have found that obese men with melanoma show better survival, and also respond better to therapy, than those who have a normal body mass index (McQuade et al., 2018). The protective effect of obesity was not seen in female melanoma patients, suggesting a possible hormonal link. It is known that fat tissue produces estrogen, and that men who are obese have higher levels of estrogen in their blood (Schneider et al., 1979). Although further work will be required to solidify the link between male obesity and GPER signaling, there is a clear role for estrogen, and particularly GPER-activating drugs, in improving outcomes for melanoma patients. If used alongside existing treatments, these drugs may in the future make established immunotherapies more effective for more patients.
